# Primates Decline Rapidly in Unprotected Forests: Evidence from a Monitoring Program with Data Constraints

**DOI:** 10.1371/journal.pone.0118330

**Published:** 2015-02-25

**Authors:** Francesco Rovero, Arafat Mtui, Amani Kitegile, Philipo Jacob, Alessandro Araldi, Simone Tenan

**Affiliations:** 1 Tropical Biodiversity Section, MUSE—Museo delle Scienze, Corso del Lavoro e della Scienza 3, Trento, Italy; 2 Udzungwa Ecological Monitoring Centre, Udzungwa Mountains National Park, Mang’ula, Tanzania; 3 College of African Wildlife Management, Mweka, Tanzania; 4 Department of Wildlife Management, Sokoine University of Agriculture, Morogoro, Tanzania; 5 Vertebrate Zoology Section, MUSE—Museo delle Scienze, Corso del Lavoro e della Scienza 3, Trento, Italy; Institute of Zoology, CHINA

## Abstract

Growing threats to primates in tropical forests make robust and long-term population abundance assessments increasingly important for conservation. Concomitantly, monitoring becomes particularly relevant in countries with primate habitat. Yet monitoring schemes in these countries often suffer from logistic constraints and/or poor rigor in data collection, and a lack of consideration of sources of bias in analysis. To address the need for feasible monitoring schemes and flexible analytical tools for robust trend estimates, we analyzed data collected by local technicians on abundance of three species of arboreal monkey in the Udzungwa Mountains of Tanzania (two *Colobus* species and one *Cercopithecus*), an area of international importance for primate endemism and conservation. We counted primate social groups along eight line transects in two forest blocks in the area, one protected and one unprotected, over a span of 11 years. We applied a recently proposed open metapopulation model to estimate abundance trends while controlling for confounding effects of observer, site, and season. Primate populations were stable in the protected forest, while the colobines, including the endemic Udzungwa red colobus, declined severely in the unprotected forest. Targeted hunting pressure at this second site is the most plausible explanation for the trend observed. The unexplained variability in detection probability among transects was greater than the variability due to observers, indicating consistency in data collection among observers. There were no significant differences in both primate abundance and detectability between wet and dry seasons, supporting the choice of sampling during the dry season only based on minimizing practical constraints. Results show that simple monitoring routines implemented by trained local technicians can effectively detect changes in primate populations in tropical countries. The hierarchical Bayesian model formulation adopted provides a flexible tool to determine temporal trends with full account for any imbalance in the data set and for imperfect detection.

## Introduction

Nonhuman primates are universally and increasingly threatened in tropical forests by habitat loss and hunting [1–4], stressing the conservation relevance of data on temporal trends in the abundance of populations [5–8]. Yet, there are relatively few long-term studies on population dynamics, largely because diurnal primates are long-living animals, hence population changes may occur over long times relatively to the duration of most studies. Indeed, systematic monitoring programs should ideally be both sustained over the medium- to long-term, and scientifically robust [9]. In turn, sustainability of monitoring efforts in countries with primate populations implies local participation (of varying intensity) in the critical task of data collection, a common and viable arrangement [10]. This approach often also applies to management-oriented decision-making by local communities or authorities based on monitoring results [10–11]. Scientific soundness, on the other hand, implies adequate capacity building to achieve standardization of sampling protocols and methodological rigor overall. However, a number of evaluations of locally-based monitoring programs has noted that this last requirement often is not met, due to detection errors in data collection and/or poor consideration, in the analysis, of potential sources of bias in the data across space, time, and data collectors [12–15].

For predominantly arboreal monkeys, the standard method for counting animals is along line transects (e.g. [16]). While the canonical approach to abundance estimation is distance sampling [17], its application to forest-dwelling primates is complex due to the poor visibility in the dense vegetation, the inherent difficulties in measuring distance to centers of primate groups that potentially include dozens of individuals, and the large sampling effort required to estimate density with statistical confidence [6,16,18,19]. Therefore, a number of long-term monitoring programs have used encounter rates with primate social groups (number of groups per km walked) as the metric of choice, given the easier data collection protocol involved [5,7,20,21]. On the other hand, the use of observed encounter rates for determining temporal trends may be of limited use, given the widely recognized importance of modeling animal counts accounting for imperfect detection (e.g. [22,23]). Indeed primate detectability can vary substantially, even irrespective of sighting distance, due to factors such as observers’ experience and ability, forest habitat type, and season [5,20,21]. In addition, the analysis of long-term and locally based monitoring efforts often involve periods of missing data. For these reasons, obtaining statistically rigorous estimates of populations trends based on primate counts remains a difficult and yet highly necessary conservation objective [6,7].

In this study, we use a recently developed modeling framework that allows the estimation of the true population trajectories and accounts for imperfect detection, to analyze count data collected by several observers over a maximum span of 11 years for three arboreal primate species in the Udzungwa Mountains of Tanzania. These are the endemic and IUCN-Endangered Udzungwa red colobus (*Procolobus gordonorum*), the Angolan black-and-white colobus (*Colobus angolensis palliatus*) and the Sykes’ monkey (*Cercopithecus mitis moloneyi/monoides*). The area is the top site in Tanzania for primate richness and endemism [24], and one of top sites for vertebrate diversity in the world [25,26]. Our aim was to determine temporal trends in the target populations by modeling primate group counts and factoring in potential sources of variation due to season and observer. A recent and complementary density estimation study in the same area has shown that group count is a valid index of abundance [19]. Target populations live in two moist forest blocks within the Udzungwa Mountains that are similar in size and habitat but contrast in protection level, one being well protected and one virtually unprotected. An earlier study on these primates, based on raw encounter rates and aimed at deciphering the relative effect of hunting and habitat degradation on populations’ relative abundance, found that populations vulnerable to hunting may be declining in the unprotected forest [4]. In particular, the study found that escalating hunting in this forest has especially affected the two colobus species, although habitat degradation may also have reduced their abundance; in contrast, the Sykes’ monkey did not show signs of decline [4]. In addition, the colobus populations in the unprotected forest are much smaller than those in the protected one, and also have a smaller group size (i.e. number of individuals; [19]). Despite the relatively long-term research effort in this ‘primate hotspot’, however, we still lack robust assessments of population trends for any primate species in the area.

## Materials and Methods

### Ethics Statement

Data collection used distance sightings of animals and hence it did not involve direct contact or interaction with the animals. Fieldwork was done under research permits (number 2006–200-NA-2006–27 and 2009–139-NA-2009–49 to FR), issued by the Tanzania Commission for Science and Technology.

### Study sites and subjects

**Fig 1:**
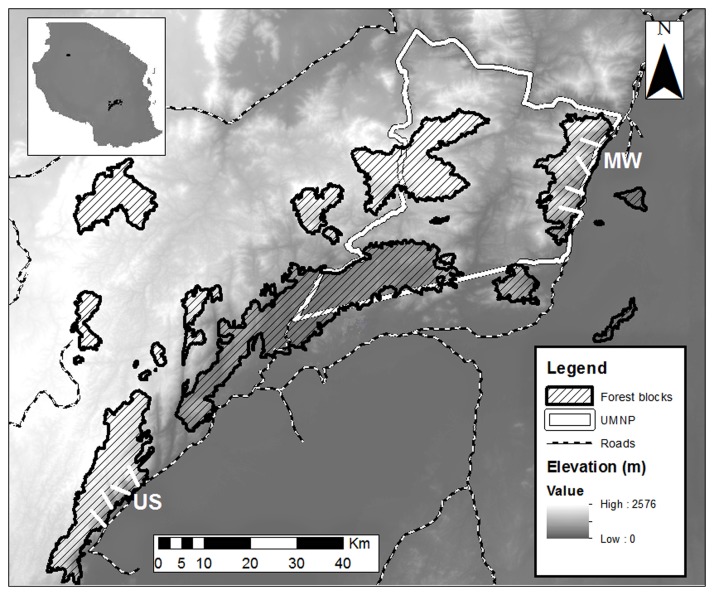
Map of the study area, the Udzungwa Mountains of Tanzania (location in the top left inset). The map shows the forest blocks among which are Mwanihana forest (MW) to the northeast and Uzungwa Scarp (US) to the southwest. The four line transects used to count primates are shown as white lines in each of these two forests. The background layer is a Digital Elevation Model (dark is lower elevation). The borders of the Udzungwa Mountains National Park (UMNP) are also indicated.

The Udzungwa Mountains of south-central Tanzania (7°40'S to 8°40'S and 35°10'E to 36°50'E; Fig. 1) are the largest mountainous block (>12 000 km^2^) in the Eastern Arc Mountains [26]. The area is a mosaic of closed forest blocks ranging in size from 12 to over 500 km^2^ and interspersed with drier habitats. Moist winds from the Indian Ocean hit primarily the east-facing slopes and trigger higher precipitation than in the surrounding areas, varying from 1500–2000 mm per year and mainly falling from December to May. We focused our studies on two of the largest forest blocks that are located on east-facing slopes: (1) Mwanihana forest (MW; 177 km^2^) in the Udzungwa Mountains National Park (UMNP), and (2) Uzungwa Scarp Forest Reserve (US, 200 km²), approximately 150 km to the southwest. These two forests have continuous forest cover at approximately 300–2000 m a.s.l. and similar habitat including zones from lowland deciduous and semi-deciduous forest to montane evergreen forest [27], but contrast dramatically in protection level. MW is relatively well protected by the statutes of the National Park, while US lacks law enforcement on the ground; hence human disturbance is very high, with both hunting and degradation known to target arboreal primates and other wildlife [4].

The three target species are the three predominantly arboreal monkey species occurring in the target forests and live in social groups that range in size from 3–83, 2–14 and 2–22 individuals per group for Udzungwa red colobus, Angolan colobus and Syke’s monkey, respectively [28]. Yellow baboon (*Papio cynocephalus*) and Sanje mangabey (*Cercocebus sanjei*) are also present in the target forests, but since they are predominantly terrestrial they are rarely sighted from line-transects [4,21]; in addition, baboons only occur along the lowland forest edge.

### Field methods

Primates were counted from 2002–2012 (with a gap in part of 2005 and in 2006; Table 1) along four line-transects in each forest. Transects consisted of regularly maintained trails predominantly oriented east to west 3.5 to 4 km in length, from forest edge to interior, i.e. sampling the lower to mid-elevation forest zones in both forests (300 to 1000 m a.s.l.). We walked along transects at least once per month by a main observer, responsible for data recording, assisted by a second observer. Five different observers were involved overall, resulting in seven different combinations of observer’ pairs (Table 1). Censuses began at 7–7:30 and observers walked at a pace of 1 km per hour. Upon sighting primate groups, the main observer annotated time, position along transect and primate species detected. Group size (i.e. the number of individuals) was not recorded due to the inherent difficulties of counting individuals in densely vegetated habitats. All observers were trained in the field in data collection procedures by F.R., who was also the only non-local observer involved.

**Table 1:** Details of line transects, sampling effort, and observers involved in counting primate groups in a protected (Mwanihana, MW) and unprotected forest (Uzungwa Scarp, US) in the Udzungwa Mountains of Tanzania during the dry season of 2002–2012.

Forest	Transect name	Lengt (km)	Sampling year (June-November)	Census replicates (km walked)	Number of different observer’ pairs
MW	Camp site 3	4.0	2002–2004, 2007–2012	156 (624.0)	4
	Mwanihana	4.0	2002–2004, 2007–2012	156 (621.8)	4
	Sanje	3.7	2002–2004, 2007–2012	154 (569.8)	4
	Msolwa	4.0	2007–2012	104 (414.7)	3
US	Ikule	3.6	2004–2005, 2007–2012	82 (283.1)	3
	Mkaraji	3.5	2004–2005, 2007–2012	81 (283.5)	4
	Jkt	3.7	2004–2005, 2007–2008	29 (107.3)	2
	Tazara	3.5	2009–2012	43 (150.5)	2

### Model formulation and parameter estimation

To analyze spatio-temporal patterns in abundance, we used a class of recently developed hierarchical models [29]. This model is a generalization of the *N*-mixture models [30] and can be applied to open populations, where abundance can change over time as a consequence of additions and deletions in the site superpopulation (i.e. the populations of animals whose home ranges overlap the sampled sites). We used this generalized model to estimate seasonal and annual group abundance of three primate species for a protected and a non-protected area, as well as trend in abundance over time. More specifically, we adopted an exponential growth model as a simple formulation of the hidden-Markov model provided by [29]. The model requires both spatial and temporal replication of surveys of unmarked individuals. Let *y*_*ijt*_ denote the number of groups of a certain species observed during the *j*th visit to the *i*th site (transect in this case) in year *t*. Primate group counts are the result of two stochastic processes, (i) the state or ecological process which describes the true local abundances (*N*_*it*_), and (ii) the observation process which links the observed counts *y*_*ijt*_ to the true abundances *N*_*it*_. The model is specified through the following distributional assumptions:

$$N_{i1}\;\sim \;Poisson\;(\lambda )$$ (eqn. 1)

$$N_{it}\;\sim \;Poisson\;(\gamma_{area}\;N_{it-1})$$ (eqn. 2)

$$\gamma_{ijt}\sim \;Binomial\;(N_{it},p_{ijt})$$ (eqn. 3)

where *λ* is the average expected abundance among all transects of two areas (MW or US) during the first year (*t* = 1), *γ*_*area*_ is the area-specific finite rate of (population) increase, and *p*_*ijt*_ is the probability of detection during the visit *j* = 1, …, *J* to transect *i* = 1, …, *R* in year *t* = 1, …, *T*. We assumed that the detectability of each species may differ among transects and observer (*obs*), as follows:

$$logit(p_{ijt})=\;\mu_{p}+\;\delta_{i}+\;\varepsilon_{obs,ijt}$$ (eqn. 4)

with *δ*_*i*_ *~ Normal*(0, *σ* ^*2*^_*transect*_) and *ε*_*obs*,*ijt*_ *~ Normal*(0, *σ* ^*2*^_*obs*_). In our case study, transects were replicated twice per month during the dry season, resulting in *J* = 12 replicates of *R* = 8 transects (four in each area), during *T* = 11 years at the most (see previous section for transect-specific sampling periods). We assumed population closure, i.e. the population (of groups) at each site remains constant, throughout the 12 replicates performed within each dry season. A minimum distance of 3 km between transects assures site independence (i.e. no groups can be detected at multiple sites).

Missing surveys for some replicates or years resulted in missing detection covariate values (observer). We properly accounted for uncertainty about the missing covariate values by specifying an underlying model for the detection covariates, i.e. by assigning a prior distribution as done for the model parameters. A sample of probable values for the missing covariates is generated by sampling new values on each iteration of the MCMC algorithm in exactly the same way that model parameters are sampled [31]. The prior for observer was set as obs_*ijt*_ ~ *Categorical*(***π***_***obs***_), where ***π***_***obs***_ is the probability vector associated to each observer, assumed drawn from a uniform distribution between 0 and 1. We chose the following priors for the other model parameters: uniform(0, 20) distribution for *λ*, uniform(-5, 5) distributions for *γ*_*area*_, uniform(0, 2) distributions for *σ* ^*2*^_*obs*_ and *σ* ^*2*^_*transect*_, and a normal(0, *σ* ^*2*^_*μ*_) distribution for *μ*_*p*_. By varying the latter variance *σ* ^*2*^_*μ*_ ∈ {10, 100, 1000} and considering an additional prior for the trend parameters *γ*_*area*_ in the form of a normal(0, 100) distribution truncated within the range (-10,10), we assessed prior sensitivity of posterior parameter estimates which remained almost the same under the six different sets of priors.

Area-specific group abundance for year *t* was estimated as ${\hat{N}}_{area,t}=\sum_{i=R_{area,min}}^{i=R_{area,\max}}N_{it}$, where *R*_*area*,*min*_ = 1 for MW and 5 for US, and *R*_*area*,*max*_ = 4 for MW and 8 for US. Since the effective area surveyed (i.e. the area over which the site superpopulation of groups resides) was unknown, we could not directly estimate density [32,33]. However, in order to compare group abundances among areas we divided annual area-specific abundances by the total length of the transects (TRL, in km) in each area, as $\frac{{\hat{N}}_{area,t}}{\sum_{i=R_{area,min}}^{i=R_{area,max}}TRL_{i}}$. To assess the adequacy of the model for each species-specific data set, we conducted a goodness-of-fit test based on the posterior predictive distribution of a Chi-squared discrepancy measure [34]. Values for Bayesian *P*-value ranging from 0.84 to 0.87 suggested an adequate fit (i.e., *P*-value was between 0.05 and 0.95). To test seasonal differences in (area-specific) group abundance and detectability, we considered a subset of two years (2011–2012) and seven transects for which counts were replicated throughout the year, once a month. Wet season (December-May) and dry season were sampled six times each (*J*_*wet*_ = *J*_*dry*_ = 6), following the methodology explained above. We tested season-specific variation in the expected abundance and detection probability by extending the model in eqn. 1–4 as follows:

$$N_{i,\;season,1}\;\sim \;Poisson\;(\lambda_{season})$$ (eqn. 5)

$$N_{i,season,t}\;\sim \;Poisson\;(\gamma_{area}\;N_{i,season,t-1})$$ (eqn. 6)

$$\gamma_{ijt}\sim \;Binomial\;(N_{i,season,t},p_{ijt})$$ (eqn. 7)

where *λ*_*season*_ is the season-specific average abundance among all transects of two areas (MW or US) for the first year (*t* = 1), and *N*_*i*,*season*,*t*_ are the true local abundances for the two seasons in year *t*. Detection probability *p* was assumed to vary in relation to transect and observer, as above, and a season-specific mean value ${\bar{p}}_{season}=expit\left(\mu_{p,season}\right)$, as follows:

$$logit(p_{ijt})=\mu_{p,wet}\;(1-x_{j})+\mu_{p,dry}\;x_{j}+\delta_{i}+\varepsilon_{obs,ijt}$$ (eqn. 8)

with *δ*_*i*_ *~ Normal*(0, *σ* ^*2*^_*transect*_) and *ε*_*obs*,*ijt*_ *~ Normal*(0, *σ* ^*2*^_*obs*_). The covariate *x*_*j*_ is an indicator of season. That is, *x*_*j*_ = 0 if counts are referred to visit *j* carried out during the wet season, and *x*_*j*_ = 1 for dry season. Note that during the selected period all surveys were carried out and the related data set did not present missing values for the detection predictor, as above. Differences in area and season-specific group abundances were evaluated by considering the average ($\bar{E}\left(N_{area,season}\right)$ hereafter) among transects and years of the related expected values, *E*(*N*_*i*,*season*,*t*_) = *γ*_*area*_ *N*_*i*,*season*,*t*-1_.

We fitted our models using a Bayesian approach together with Markov chain Monte Carlo simulation methods [35]. Summaries of the posterior distribution were calculated from three independent Markov chains initialized with random starting values, run 110,000 times after a 10,000 iteration burn-in and re-sampling every 20 draws. We assessed convergence of chains to their stationary distributions using the Brooks-Gelman-Rubin convergence diagnostic [36]. Models were implemented in program JAGS [37], that we executed using R [38] with the packages *rjags* [39], *dclone* [40], and *snow* [41]. An R script with the BUGS single-season model specification is provided in S2 File.

## Results

**Fig 2:**
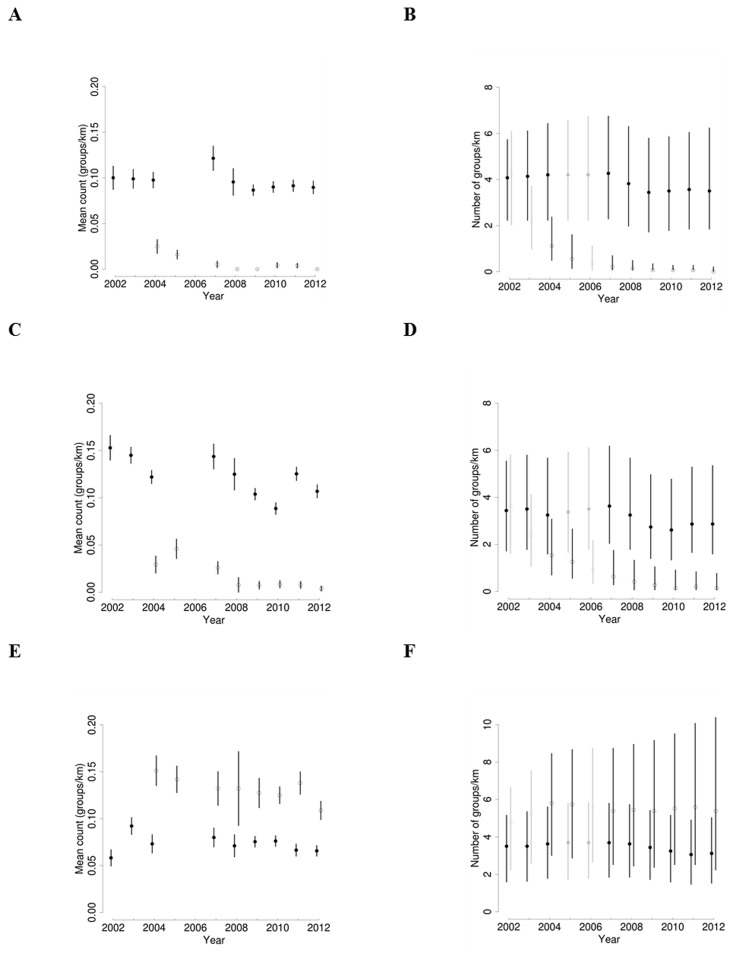
Results of observed and estimated primate abundance from line transect counts in the Udzungwa Mountains of Tanzania. Number of primate groups per km of transect at Mwanihana (solid symbols) and Uzungwa Scarp (open symbols) for Angolan colobus (A,B), Udzungwa red colobus (C,D), and Sykes' monkey (E,F). (A), (C), and (E) show the mean observed counts per km (with 1 SE); (B), (D), and (F) show the estimated total number of groups per km (posterior median and 95% CRI). The predicted values for years with no surveys are shown in gray.

A total of 805 census walks were conducted along eight transects (four in each of the two areas) during the dry season of 11 years (2002–2012, Table 1). Primate counts were available from 65% of all site-year combinations, and individual transects were surveyed for 3–9 years (median 8). Group counts ranged from 0 to 6 (median 1) for all species (Fig. 2a, c, and e).

**Fig 3:**
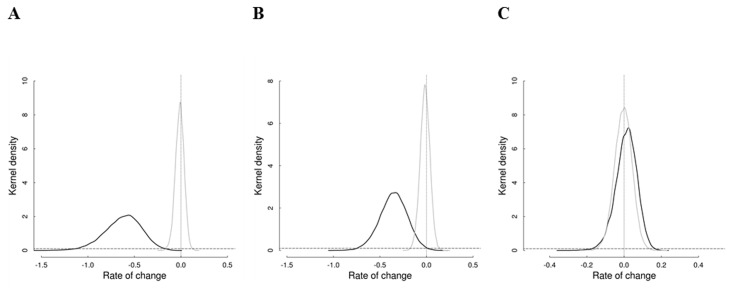
Prior (dashed line) and posterior (solid lines) distributions of the parameters for abundance trends, referred to Mwanihana (*γ*_*MW*_, gray solid line) and Uzungwa Scarp (*γ*_*US*_, black solid line). Results are for (A) Angolan colobus, (B) Udzungwa red colobus, and (C) Sykes' monkey. A uniform (-5, 5) prior is used in all cases. The vertical dotted line indicates population stability.

By correcting group abundance for heterogeneity in detection probability within the hierarchical model, we estimated trend coefficients on average close to zero (i.e. group population stability) for all species in the protected, Mwanihana forest (Angolan colobus *γ*_*MW*_ = -0.013, -0.110–0.076, 95% credible interval; Udzungwa red colobus-0.016, -0.121–0.086; Sykes' monkey-0.005, -0.100–0.086; Table 2, Fig. 2b, d, and f). In contrast, in the unprotected Uzungwa scarp the number of groups decreased, on average, for both Angolan colobus (*γ*_*US*_ = -0.624, -1.048–-0.270) and Udzungwa red colobus (-0.355, -0.657–0.076), while remained stable for Sykes' monkey (0.011, -0.111–0.116; Fig. 3).

**Table 2:** Posterior summary of model parameters for count data of three primate species (Angolan colobus, Udzungwa red colobus, and Sykes' monkey) for a protected (Mwanihana, MW) and a non protected forest (Uzungwa scarp, US) in Tanzania.

Species	Parameter	Posterior mean	Posterior SD	2.5%	97.5%
Angolan colobus	*λ*	15.299	3.280	8.109	19.814
	*γMW*	-0.013	0.047	-0.110	0.076
	*γUS*	-0.624	0.198	-1.048	-0.270
	*$\bar{p}$*	0.109	0.041	0.049	0.213
	*σtransect*	0.416	0.419	0.010	1.604
	*σobs*	0.158	0.180	0.005	0.640
Udzungwa red colobus	*λ*	13.336	3.808	6.376	19.636
	*γMW*	-0.016	0.052	-0.121	0.086
	*γUS*	-0.355	0.148	-0.657	-0.076
	*$\bar{p}$*	0.153	0.058	0.067	0.288
	*σtransect*	0.509	0.410	0.020	1.563
	*σobs*	0.248	0.231	0.008	0.854
Sykes' monkey	*λ*	15.700	3.204	8.425	19.851
	*γMW*	-0.005	0.047	-0.100	0.086
	*γUS*	0.011	0.058	-0.111	0.116
	*$\bar{p}$*	0.091	0.035	0.048	0.179
	*σtransect*	0.362	0.294	0.015	1.106
	*σobs*	0.172	0.155	0.007	0.561

Mean, SD, 2.5% and 97.5% percentiles are reported for each parameter.

*λ* denotes average expected abundance (during the first year) among all transects of the two areas, *γ*_*MW*_ and *γ*_*US*_ are the independent population (of groups) changing rates at each area, $\bar{p}$ is the mean detection probability in probability scale (i.e. $\bar{p}=expit\left(\mu_{p}\right)$), *σ*_*transect*_ is the standard deviation for the unexplained variability among transects, and *σ*_*obs*_ is the observer standard deviation (the latter two parameters are in logit scale).

Mean detection probability $\bar{p}$ ranged from 0.091 (0.048–0.179) for Sykes' monkey to 0.153 (0.067–0.288) for Udzungwa red colobus. Heterogeneity in detection probability *p* was apparently caused more by transect-specific variability (median values for *σ*_*transect*_ ranged from 0.362 in Sykes' monkey to 0.509 in Udzungwa red colobus), than by differences among observers (median values for *σ*_*obs*_ ranged from 0.158 in Angolan colobus to 0.248 in Udzungwa red colobus; Table 2). Estimates for season-specific parameters did not reveal any substantial differences between wet and dry season in both area-specific expected abundances ($\bar{E}\left(N_{area,season}\right)$) and detection probabilities (${\bar{p}}_{wet}$, ${\bar{p}}_{dry}$; Table A and Figure A in S1 File).

## Discussion

Our study shows that primate counts collected by local and trained field technicians and using a simple, standardized transect-based field protocol provide data that can be analyzed within a robust statistical framework to determine temporal trends of population abundance. The main result we found on population trends is that estimated group abundance appears stable for all species in the protected Mwanihana forest (MW), while in the unprotected Uzungwa Scarp (US) forest there was a marked decline for both colobus monkey species but not for the Sykes’ monkey. This finding agrees with previous analysis of part of the data using raw encounter rates [4] as well as the more recent and forest-wide abundance estimation study [19]. Decline of colobus in US is best explained by increasing human disturbance, in the form of both targeted hunting pressure by the local tribe for subsistence and habitat degradation mainly through tree and pole cutting [4]. While decoupling the relative effect on primate abundance of these two types of human pressure is complex, Rovero et al. [4] raised clear evidence of intense and escalating hunting in US by members of the local tribe using dogs to drive monkeys on isolated canopy trees for easier shooting with shotguns; this practice targets specifically the colobus monkeys which are typically arboreal and live in large groups. Our results therefore confirm the detrimental effect of hunting on the abundance of these primates, in line with findings from other studies (e.g. [42–44]). Sykes’ monkeys, on the contrary, are seemingly highly resilient to disturbance and capable of sustaining themselves in secondary, degraded forests which dominate part of the areas sampled by the study transects [4,21].

The second important result we obtained is that, despite a number of different observers were involved in data collection, the unexplained variability in detection probability among transects is greater than the variability due to observers. This is of critical importance, as it indicates that the simple field training given to all field technicians in transect walk procedures, species identification and recording of observations is adequate to collect consistent data. Third, the lack of seasonal differences in both primate abundance and detectability supports the concentration of sampling in dry seasons for future studies. Monitoring in the dry season is logistically easier and is considerably more cost-efficient than monitoring throughout the year, because walking transects during the rainy season involves higher risk of failure due to frequent rains, in addition to poorer road conditions, making access to remote forests more difficult. While the modelling approach we adopted does not require data collected in a robust design, and it can be used even if only one sample is available for each primary period (season), the availability of replicates within a season improves accuracy and precision in parameter estimates.

We assumed primate group count, our raw metric of abundance, to be positively related to true population abundance, as shown by a complementary, abundance estimation study that used systematic distance sampling in the same area ([19]; see also [45]). Hence, temporal changes in this metric will indicate population changes at these sites. However, since the sampling areas associated with each transect remain unknown, our estimated, transect-based abundance is still effectively a relative population abundance estimate [32]. In addition, we cannot exclude that trail maintenance (which is necessary given the long duration of the monitoring program) may have induced variations in primate abundance relative to other parts of the forest by facilitating access to hunters or people cutting poles and trees. Consequently, our inference on trends in abundance would be limited to areas near maintained transects. Our spatial design of transects was a compromise between site accessibility (i.e. ease of reaching the transect start from the road allowing maintenance of transects over the years), and adequate sampling coverage in the forest zones where primate monitoring is most informative and conservation-relevant. This is because the low-to-medium elevation zones in both forests are the most encroached ones due to the adjacent high human density [4]. Meanwhile, all three target species are found in greater abundance in these forest habitat types (i.e. from lowland deciduous to sub-montane evergreen) than at higher elevation, evergreen montane forest [19,46]. For these reasons, we are confident that the temporal trends we obtained provide adequate indications about the conservation status of these threatened primate populations.

Our choice of using counts of social groups instead of counts of individuals was guided by the proven difficulties of counting individuals with confidence in the dense forests in the Udzungwa Mountains [16,19]. This choice has two implications. First, because group size can change considerably across forests [47], population-specific demographic data are relevant to interpreting differences in abundance between MW and US. The unprotected US has smaller group size than MW, reflecting a pattern of altered population demography considered an effect of human disturbance [19,47]. Therefore, the differences we found in estimated group abundance between US and MW would be even larger if individual abundance would be considered, with proportionally lower abundance in US. Second, group size may change with time within a population, hence the rate of change in group abundance may differ from that of individual abundance. In our case, the documented, increasing disturbance in US relative to MW [4] likely implies that group size have concordantly decreased. However, since group size may affect group detectability [18–19] a decreasing group size with time, such as the one that potentially occurred in US, would result in a less negative trend in group abundance than we estimated. In view of these considerations, group size data taken periodically in the course of the monitoring program are critically important to scale trends (see [48]).

### Conclusions and conservation recommendations

Our study shows that simple and locally based monitoring routines can effectively detect changes in primate population numbers in remote areas, hence providing scientifically-sound and critical information on the conservation status of target species. We envisage that continued, longer-term data will be needed to accurately assess population trends, given the natural oscillations of primate populations noted by the few long-term studies available, and the potentially long delay between perturbation events and population responses (e.g. [5,6]). Yet, our data represent the longest data-set on any biological component of the Udzungwa Mountains, an outstanding biodiversity hotspot, and the results clearly point to a critical situation for the viability of colobus monkeys, including the endemic red colobus, in Uzungwa scarp. This forest has long been known as one of the most important and yet most threatened in the area [49]. Unless effective protection is urgently applied, local extinction of these populations may occur. On the positive side, our results have brought this critical situation to the attention of the Tanzania Government [50] and, as a result, the forest is currently being upgraded to Nature Reserve status (N. Burgess, pers. comm.), a rare example of local decision-making based on monitoring data [10]. Indeed our study stresses the importance of locally based monitoring beyond its simplest configuration of local involvement limited to data collection.

## Supporting Information

S1 File Summaries of model parameters for seasonal count data of primates (Table A), area- and season-specific averages of the expected abundance, and mean seasonal detection probabilities (Figure A). (PDF)

S2 File R and JAGS code for the single-season models. (R)

## References

[1] Cowlishaw G, Dunbar R. Primate Conservation Biology. Chicago: University of Chicago Press; 2000.

[2] Harcourt AH, Doherty DA. Species-area relationships of primates in tropical forest fragments: a global analysis. J Appl Ecol. 2005;42: 630–637.

[3] Linder JM, Oates JF. Differential impact of bushmeat hunting on monkey species and implications for primate conservation in Korup National Park, Cameroon. Biol Conserv. 2011;144: 738–745.

[4] Rovero F, Mtui A, Kitegile A, Nielsen M. Hunting or habitat degradation? Decline of primate populations in Udzungwa Mountains, Tanzania: An analysis of threats. Biol Conserv. 2012;146: 89–96.

[5] Chapman CA, Balcomb SR, Gillespie TR, Skorupa JP, Struhsaker TT. Long-term effects of logging on African primate communities: A 28-year comparison. Conserv Biol. 2000;14: 207–217.

[6] Chapman CA, Struhsaker TT, Skorupa JP, Snaith TV, Rothman JM. Understanding long-term primate community dynamics: implications for forest change. Ecol Appl. 2010;20: 179–191. 20349839 10.1890/09-0128.1

[7] Lwanga JS, Struhsaker TT, Struhsaker PJ, Butynski TM, Mitani JC. Primate population dynamics over 32.9 years at Ngogo, Kibale National Park, Uganda. Am J Primatol. 2011;15: 1–15. 10.1002/ajp.20965 21557287

[8] N’Goran PK, Boesch C, Mundry R, N’Goran EK, Herbinger I, Yapi FA, et al Hunting, law enforcement, and African primate conservation. Conserv Biol. 2012;26: 565–571. 10.1111/j.1523-1739.2012.01821.x 22394275

[9] Danielsen F, Burgess ND, Balmford A. Monitoring matters: examining the potential of locally based approaches. Biodivers Conserv. 2005;14: 2507–2542.

[10] Danielsen F, Burgess ND, Balmford A, Donald PF, Funder M, Jones JP, et al Local participation in natural resource monitoring: a characterization of approaches. Conserv Biol. 2009;23: 31–42. 10.1111/j.1523-1739.2008.01063.x 18798859

[11] Andrianandrasana HT, Randriamahefasoa J, Durbin J, Lewis RE, Ratsimbazafy JH. Participatory ecological monitoring of the Alaotra wetland in Madagascar. Biodivers Conserv. 2005;14: 2757–2774.

[12] Yoccoz NG, Nichols JD, Boulinier T. Monitoring of biological diversity in space and time. Trends Ecol Evol. 2001;16: 446–453.

[13] Pollock KH, Nichols JD, Simons TR, Farnsworth GL, Bailey LL, Sauer JR. Large scale wildlife monitoring studies: statistical methods for design and analysis. Environmetrics. 2002;13: 105–119.

[14] Keane A, Jones JPG, Milner-Gulland EJ. Encounter data in resource management and ecology: pitfalls and possibilities. J Appl Ecol. 2011;48: 1164–1173.

[15] Burton AC. Critical evaluation of a long-term, locally based wildlife monitoring program in West Africa. Biodivers Conserv. 2012;21: 3079–3094.

[16] Marshall AR, Lovett JC, White PCL. Selection of line-transect methods for estimating the density of group-living animals: lessons from the primates. Am J Primatol. 2008;70: 452–462. 10.1002/ajp.20516 18240143

[17] Buckland ST, Anderson DR, Burnham KP, Laake JL, Borchers DL, Thomas L. Introduction to Distance Sampling: Estimating Abundance of Biological Populations. Oxford: Oxford University Press; 2001

[18] Buckland ST, Plumptre AJ, Thomas L, Rexstad E. Design and analysis of line transect surveys for primates. Int J Primatol. 2010;31: 833–847.

[19] Araldi A, Barelli C, Hodges K, Rovero F. Density estimation of the endangered Udzungwa red colobus (*Procolobus gordonorum*) and other arboreal primates in the Udzungwa Mountains using systematic distance sampling. Int J Primatol. 2014;35: 941–956.

[20] Mitani JC, Struhsaker TT, Lwanga JS. Primate Community Dynamics in Old Growth Forest over 23.5 Years at Ngogo, Kibale National Park, Uganda: Implications for Conservation and Census Methods. Int J Primatol. 2000;21: 269–286.

[21] Rovero F, Struhsaker TT, Marshall AR, Rynne TA, Pedersen UB, Butynski TM, et al Abundance of Diurnal Primates in Mwanihana Forest, Udzungwa Mountains, Tanzania. Int J Primatol. 2006;27: 675–697.

[22] Williams BK, Nichols JD, Conroy MJ. Analysis and management of animal populations. San Diego: Academic Press; 2002.

[23] Royle JA, Dorazio R. Hierarchical modeling and inference in ecology: the analysis of data from populations, metapopulations and communities. Academic Press; 2008.

[24] Davenport TRB, Nowak K, Perkin A. Priority primate areas in Tanzania. Oryx. 2013;48: 39–51.

[25] Le Saout S, Hoffmann M, Shi Y, Hughes A, Bernard C, Brooks TM. Protected Areas and Effective Biodiversity Conservation. Science. 2013;342: 803–805. 10.1126/science.1239268 24233709

[26] Rovero F, Menegon M, Fjeldså J, Collett L, Doggart N, Leonard C, et al Targeted vertebrate surveys enhance the faunal importance and improve explanatory models within the Eastern Arc Mountains of Kenya and Tanzania. Divers Distrib. 2014;20: 1438–1449.

[27] Lovett JC, Marshall AR, Carr J. Changes in tropical forest vegetation along an altitudinal gradient in the Udzungwa Mountains National Park, Tanzania. Afr J Ecol. 2006;44: 478–490.

[28] Rovero F, Marshall AR, Jones T, Perkin A. The primates of the Udzungwa Mountains: diversity, ecology and conservation. J Anthropol Sci. 2009;87: 93–126. 19663171

[29] Dail D, Madsen L. Models for estimating abundance from repeated counts of an open metapopulation. Biometrics. 2011;67: 577–587. 10.1111/j.1541-0420.2010.01465.x 20662829

[30] Royle JA. N-Mixture Models for Estimating Population Size from Spatially Replicated Counts. Biometrics. 2004;60: 108–115. 15032780 10.1111/j.0006-341X.2004.00142.x

[31] Gimenez O, Bonner SJ, King R, Parker RA, Brooks SP, Jamieson LE, et al WinBUGS for Population Ecologists: Bayesian Modeling Using Markov Chain Monte Carlo Methods In: Thomson DL, Cooch EG, Conroy MJ, editors. Modeling demographic processes in marked populations. Environmental and Ecological Statistics. New York: Springer-Verlag; 2009 pp. 883–915.

[32] Chandler RB, Royle JA, King DI. Inference about density and temporary emigration in unmarked populations. Ecology. 2011;92: 1429–1435. 21870617 10.1890/10-2433.1

[33] Chandler RB, King DI. Habitat quality and habitat selection of golden-winged warblers in Costa Rica: an application of hierarchical models for open populations. J Appl Ecol. 2011; 48: 1038–1047.

[34] Gelman A, Carlin JB, Stern HS, Rubin DB. Bayesian Data Analysis. Boca Raton: Chapman and Hall; 2004.

[35] Brooks SP. Bayesian computation: a statistical revolution. Philos Trans A Math Phys Eng Sci A. 2003;361: 2681–2697. 14667292 10.1098/rsta.2003.1263

[36] Brooks SP, Gelman A. Alternative methods for monitoring convergence of iterative simulations. J Comput Graph Stat. 1998;7: 434–455.

[37] Plummer M. JAGS: A program for analysis of Bayesian graphical models using Gibbs sampling. Proceedings of the 3rd International Workshop on Distributed Statistical Computing; 2003. Available: http://citeseerx.ist.psu.edu/viewdoc/summary?doi=10.1.1.13.3406. Accessed 2014 Aug 4.

[38] R Development Core Team. R: A Language and Environment for Statistical Computing. R Foundation for Statistical Computing, Vienna, Austria; 2013 Available: http://www.R-project-org. Accessed 2014 Aug 4.

[39] Plummer M. Rjags: Bayesian graphical models using MCMC. R package version 3.10; 2013. Available: http://CRAN.R-project.org/package=rjags. Accessed 2014 Aug 4.

[40] Sòlymos P. Dclone: Data Cloning in R. The R Journal 2: 29–37; 2010 Available: http://journal.r-project.org. Accessed 2014 Aug 4.

[41] Tierney L, Rossini AJ, Li N, Sevcikova H. Now: Simple Network of Workstations. R package version 0.3–12; 2013. Available: http://CRAN.R-project.org/package=snow. Accessed 2014 Aug 4.

[42] Oates JF. Habitat alteration, hunting and the conservation of folivorous primates in African forests. Aust J Ecol 1996;21: 1–9.

[43] de Thoisy B, Renoux F, Julliot C. Hunting in northern French Guiana and its impact on primate communities. Oryx. 2005;39: 149–159.

[44] Linder JM, Oates JF. Differential impact of bushmeat hunting on monkey species and implications for primate conservation in Korup National Park, Cameroon. Biol Conserv. 2011;144: 738–745.

[45] Seber G. The estimation of animal abundance and related parameters. New York: Macmillan; 1982

[46] Barelli C, Gallardo Palacios JF, Rovero F. Variation in primate abundance along an elevational gradient in the Udzungwa Mountains of Tanzania In: Grow NB, Gursky-Doyen S, Krzton A, editors. High Altitude Primates. New York: Springer; 2014 pp. 211–226.

[47] Struhsaker TT, Marshall AR, Detwiler KM, Siex K, Ehardt CL, Lisbjerg DD, et al Demographic variation among the Udzungwa Red colobus (*Procolobus gordonorum*) in relation to gross ecological and sociological Parameters. Int J Primatol. 2004;25: 615–658.

[48] Langtimm CA, Dorazio RM, Stith BM, Doyle TJ. New aerial survey and hierarchical model to estimate manatee abundance. J Wildl Manage. 2011;75: 399–412.

[49] Dinesen L, Lehmberg T, Rahner MC, Fjeldså J. Conservation priorities for the forests of the Udzungwa Mountains, Tanzania, based on primates, duikers and birds. Biol Conserv. 2001;99: 223–236.

[50] Rovero F, Mtui A, Kitegile A, Nielsen M, Jones T. Uzungwa Scarp Forest Reserve in crisis. An urgent call to protect one of Tanzania’s most important forests. Dar es Salaam; 2010. Available: http://www.tfcg.org/pdf/USFRReportFINALHighRes.pdf. Accessed 2014 Aug 4.

