# Supplementary material for: Primates Decline Rapidly in Unprotected Forests: Evidence from a Monitoring Program with Data Constraints
Source: PLoS One. 2015 Feb 25;10(2):e0118330. doi: 10.1371/journal.pone.0118330 (PMC4340938; doi:10.1371/journal.pone.0118330)
Supplement: S1 File — (PDF) [file pone.0118330.s001.pdf]

Table A. Posterior summaries of model parameters, under the model formulated in eqn. 5-8 for seasonal count data of three primate species (Angolan colobus, Udzungwa red colobus, and Sykes' monkey) for a protected (Mwanihana, MW) and a non protected area (Uzungwa scarp, US) in Tanzania. Data were gathered during wet and dry season (December-May, June-November respectively) in 2011-2012. Mean, SD, 2.5% and 97.5% percentiles are reported for each parameter.

Species	Parameter	Posterior mean	Posterior SD	2.5%	97.5%
Angolan colobus	λ_{wet}	11.062	5.178	2.403	19.560
	λ_{dry}	11.947	4.738	3.659	19.594
	$\bar{p}_{wet}$	0.092	0.090	0.012	0.355
	$\bar{p}_{dry}$	0.113	0.093	0.018	0.372
	$\bar{E}(N_{MW,wet})/km$	1.456	0.943	0.314	3.808
	$\bar{E}(N_{MW,dry})/km$	1.633	0.968	0.431	4.022
	$\bar{E}(N_{US,wet})/km$	0.274	0.665	0.006	1.749
	$\bar{E}(N_{US,dry})/km$	0.279	0.688	0.006	1.801
	γ_{MW}	0.650	0.380	-0.046	1.410
	γ_{US}	-3.104	1.258	-4.917	-0.420
	$\sigma_{transect}$	0.785	0.476	0.051	1.839
	σ_{obs}	0.708	0.500	0.036	1.838

Udzungwa red colobus	λ_{wet}	9.817	4.808	2.998	19.304
	λ_{dry}	11.678	4.544	4.111	19.543
	$\bar{p}_{wet}$	0.161	0.121	0.024	0.471
	$\bar{p}_{dry}$	0.139	0.101	0.022	0.410
	$\bar{E}(N_{MW,wet})/km$	0.639	0.381	0.199	1.576
	$\bar{E}(N_{MW,dry})/km$	0.740	0.369	0.264	1.652
	$\bar{E}(N_{US,wet})/km$	0.992	1.270	0.053	4.282
	$\bar{E}(N_{US,dry})/km$	1.360	1.518	0.094	5.332
	γ_{MW}	-0.044	0.323	-0.750	0.542
	γ_{US}	-1.050	0.894	-3.034	0.527
	$\sigma_{transect}$	0.877	0.438	0.153	1.843
	σ_{obs}	0.678	0.519	0.025	1.853
Sykes' monkey	λ_{wet}	9.454	4.663	3.210	19.202
	λ_{dry}	12.726	4.331	4.795	19.640
	$\bar{p}_{wet}$	0.220	0.134	0.048	0.534
	$\bar{p}_{dry}$	0.138	0.084	0.038	0.359
	$\bar{E}(N_{MW,wet})/km$	0.416	0.308	0.100	1.214

$\bar{E}(N_{MW,dry})/km$	0.569	0.312	0.169	1.349
$\bar{E}(N_{US,wet})/km$	2.156	1.232	0.647	5.187
$\bar{E}(N_{US,dry})/km$	2.895	1.337	0.849	5.911
γ_{MW}	-0.360	0.427	-1.307	0.391
γ_{US}	-0.161	0.219	-0.616	0.248
$\sigma_{transect}$	0.482	0.310	0.034	1.259
σ_{obs}	0.582	0.478	0.021	1.773

λ_{wet} and λ_{dry} denote the average expected abundance (during the first year) among all transects of the two areas, $\bar{p}_{wet}$ and $\bar{p}_{dry}$ are the mean seasonal detection probabilities in probability scale (i.e. $\bar{p}_{season} = expit(\mu_{p,season})$), $\bar{E}(N_{area,season})/km$ is the area and season-specific average (among transects and years) of the expected abundance values $E(N_{i,season,t}) = \gamma_{area} N_{i,season,t-1}$ (reported as expected number of groups/km), γ_{MW} and γ_{US} are the independent population changing rates at each area, $\sigma_{transect}$ is the standard deviation for the unexplained variability among transects, and σ_{obs} is the observer standard deviation (the latter two parameters are in logit scale).

Figure A. Area and season-specific average (among transects and years) of the expected abundance values $E(N_{i,season,t}) = \gamma_{area} N_{i,season,t-1}$, reported as expected number of groups/km (noted as $\bar{E}(N_{area,season})/km$) for (a) Angolan colobus, (c) Udzungwa red colobus, and (e) Sykes' monkey. For the same species the mean seasonal detection probabilities in probability scale (i.e. $\bar{p}_{season} = expit(\mu_{p,season})$) are also reported (b,d,f).

a)

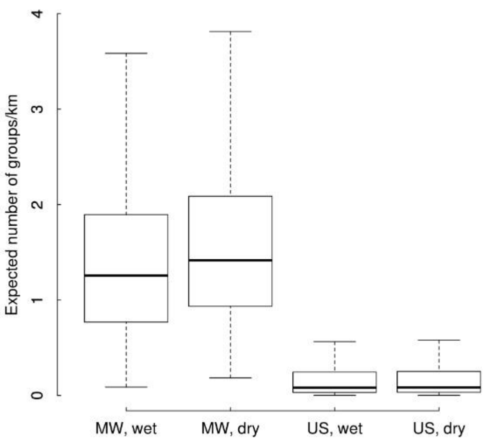

b)

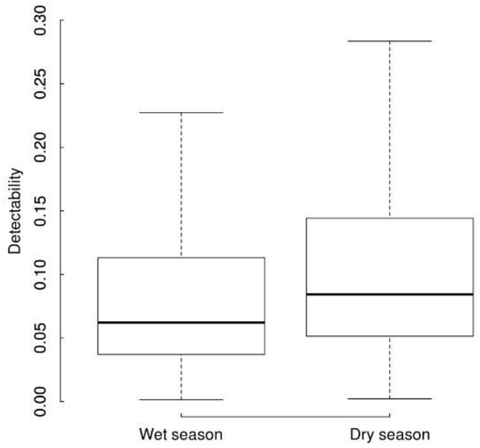

c)

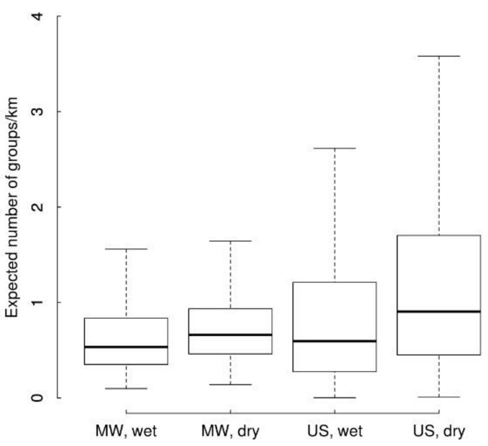

d)

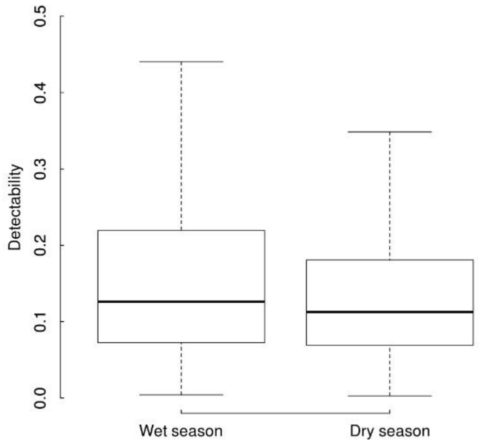

e)

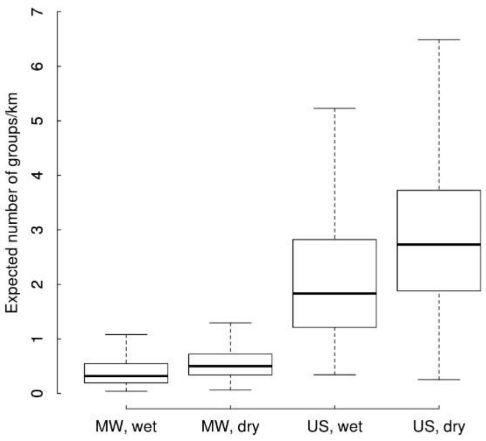
Expected number of groups/km
0
1
2
3
4
5
6
7
MW, wet
MW, dry
US, wet
US, dry

f)

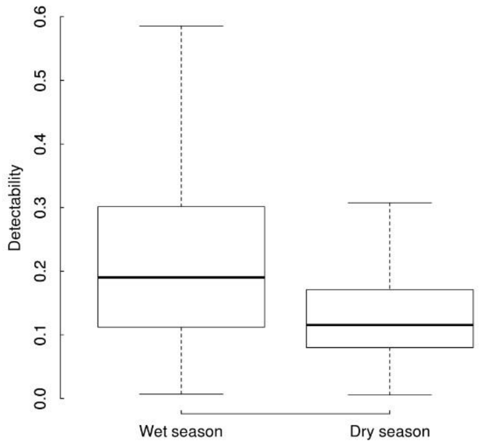
Detectability
0.0
0.1
0.2
0.3
0.4
0.5
0.6
Wet season
Dry season
